# Chloroplast genome‐based genetic resources via genome skimming for the subalpine forests of Japan and adjacent regions

**DOI:** 10.1002/ece3.11584

**Published:** 2024-07-17

**Authors:** James R. P. Worth, Satoshi Kikuchi, Seiichi Kanetani, Daiki Takahashi, Mineaki Aizawa, Elena A. Marchuk, Hyeok Jae Choi, Maria A. Polezhaeva, Viktor V. Sheiko, Saneyoshi Ueno

**Affiliations:** ^1^ Department of Forest Molecular Genetics and Biotechnology Forestry and Forest Products Research Institute, Forest Research and Management Organization Tsukuba Ibaraki Japan; ^2^ Hokkaido Research Centre Forestry and Forest Products Research Institute, Forest Research and Management Organization Sapporo Hokkaido Japan; ^3^ Kyushu Research Center Forestry and Forest Products Research Institute Kumamoto Japan; ^4^ Kawatabi Field Science Centre, Graduate School of Agricultural Science Tohoku University Sendai Miyagi Japan; ^5^ Department of Forest Science, School of Agriculture Utsunomiya University Utsunomiya Tochigi Japan; ^6^ Botanical Garden‐Institute of the Far Eastern Branch of the Russian Academy of Sciences Vladivostok Russia; ^7^ Department of Biology & Chemistry College of Natural Sciences, Changwon National University Changwon Korea; ^8^ Institute of Plant and Animal Ecology Russian Academy of Sciences Yekaterinburg Russia; ^9^ Sakhalin Branch of the Botanical Garden‐Institute FEB RAS Yuzhno‐Sakhalinsk Russia

**Keywords:** angiosperms, boreal forests, chloroplast assembly, conifers, mutational hotspots, ultra‐barcode

## Abstract

The Japanese subalpine zone is dominated by an ecologically important forest biome, subalpine coniferous forest, constituting a distinct assemblage of cold‐tolerant angiosperm and conifer species. While being relatively intact compared to other forest biomes in Japan, subalpine coniferous forests are under significant threat from deer browsing, global warming and small population size effects. However, there is a severe lack of genetic resources available for this biome's major constituent plant species. This study aimed to develop chloroplast genome‐based genetic resources for 12 widespread subalpine tree and shrub species (7 angiosperms and 5 conifers) via genome skimming of whole‐genomic DNA using short reads (100–150 bp in length). For 10 species, whole chloroplast genomes were assembled via de novo‐based methods from 4 to 10 individuals per species sampled from across their ranges in Japan and, for non‐Japanese endemic species, elsewhere in northeast Asia. A total of 566 single nucleotide polymorphisms for Japanese samples and 768 for all samples (varying from 2 to 202 per species) were identified which were distributed in geographically restricted lineages in most species. In addition, between 9 and 58 polymorphic simple sequence repeat regions were identified per species. For two Ericaceae species (*Rhododendron brachycarpum* and *Vaccinium vitis‐idaea*) characterised by large chloroplast genomes, de novo assembly failed, but single nucleotide polymorphisms could be identified using reference mapping. These data will be useful for genetic studies of species taxonomic relationships, investigating phylogeographic patterns within species, developing chloroplast‐based markers for conservation genetic studies and has potential application for studies of environmental and ancient DNA.

## Graphical abstract

In this study, we assemble 92 whole chloroplast genomes from 12 subalpine plant species (5 conifers and 7 angiosperms) and identify intraspecific variation and lineages that will be useful for evolutionary, phylogeographic and conservation‐focused studies of the threatened subalpine forest biome of Japan and adjacent regions in northeast Asia.

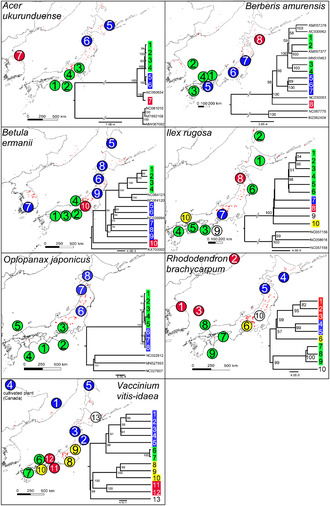

## 1 INTRODUCTION

Genome skimming is the shallow sequencing of genomic DNA enabling the accurate sequencing of genomic components such as the chloroplast, mitochondria, and high‐copy nuclear regions (Hollingsworth et al., 2016). In the last decade, genome skimming has revolutionised the ease with which genomic data can be obtained for both model and non‐model species (Dodsworth, 2015). For example, the ability to cost‐effectively and reliably assemble the relatively small mitochondria of animals and chloroplast genome of plants using de novo or reference mapping methods from low‐coverage genome skimming data has accelerated our understanding of the variation in size, gene content and arrangement of these genomes (Li et al., 2020) with thousands of whole organelle genomes now available on GenBank. Whole mitochondria (De Mandal et al., 2014) and chloroplast (Song et al., 2023) genomes are now being incorporated into barcode libraries (called ultra‐barcodes) providing greater power to understand the phylogenetic affinity of taxa and increasing the ability to identify the small fragments of organelle DNA derived from ancient or environmental samples. The relatively low cost of genome skimming means that the whole organelle genome of multiple samples can feasibly be obtained, facilitating the discovery of potentially phylogenetically informative characters and mutational hotspots for the taxonomic level of interest (e.g. genus or species level). In plants, such studies have increased in recent years identifying informative chloroplast DNA variation for phylogenetic (Fu et al., 2022; Malé et al., 2014), phylogeographic (Migliore et al., 2020) and conservation genetic (Worth et al., 2023) studies.

Here, we use genome skimming of genomic DNA to develop chloroplast genome genetic resources for an important but threatened forest biome in Japan, subalpine coniferous forests. These forests, classified in the Abieti‐Piceetalia jesoensis order (Krestov & Omelko, 2010), are an outlier of the extensive boreal forests of Eurasia. In Japan, they have a wide but fragmented distribution from northern Hokkaido (45.4° N) to the highest peaks of the Kii Peninsula (34.1° N) and Shikoku Island (33.7° N) in western Japan. The fossil record shows that subalpine forests were more widespread during glacial periods, occurring more than a thousand metres below their current elevational lower limit to near sea level in Honshu. However, during warm interglacials, including the present Holocene, their range contracted sometimes involving significant range losses of individual species, especially in western Japan (e.g. the loss of *Tsuga diversifolia* (Maxim.) Mast.) from the Chugoku region (Takahara et al., 2001), *Pinus koraiensis* Siebold et Zucc from most of western Japan (Aizawa et al., 2012) and *Picea maximowiczii* Regel ex Mast. from Kyushu and Chugoku (Magri et al., 2020).

Japan's subalpine coniferous forests are of immense value for biodiversity, harbouring many endemic species, and for the ecosystem services they provide, for example, via their role as the headwater forests of Japan's major watersheds. Although significant areas of subalpine forests have been replaced by Japanese larch (*Larix kaempferi* (Lamb.) Carrière) plantations (Franklin et al., 1979), the biome is comparatively one of the least disturbed by humans. However, despite this, these forests face significant threats including severe browsing by increasing populations of deer, small population size effects and rising global temperatures (Oishi & Doei, 2015; Tsuyama et al., 2015). Subalpine coniferous forests are considered most at risk of decline in western Japan where they have little available higher ground to migrate to, already being restricted to near the tops (Hämet‐Ahti et al., 1974) of relatively low mountain ranges. Indeed, populations of many species of subalpine trees and shrubs in western Japan are endangered (Japanese Red Data Book online: http://jpnrdb.com/), especially on Shikoku Island and the Kii Peninsula.

Genetic markers can inform conservation management of plants by resolving taxonomic uncertainty of species, identifying populations that should be prioritised for conservation based on their genetic distinctiveness (Shibabayashi et al., 2023) and revealing areas with significantly high or low genetic diversity. However, there are limited genetic markers available for the component species of Japan's subalpine forests. The existing range‐wide organelle‐based genetic studies (*Abies veitchii* (Uchiyama et al., 2023), *Picea jezoensis* (Aizawa et al., 2007), *Pinus koraiensis* (Aizawa et al., 2012) and *Vaccinium vitis‐idea* (Ikeda et al., 2015)) all are based on Sanger sequencing of a few chloroplast fragments. In this study, we develop genetic resources based on whole chloroplast genome sequencing via genome skimming for 12 trees and shrubs (Table 1) that are key components of Japan's subalpine forests occurring either as canopy dominants or important in the understory (Franklin et al., 1979; Sugita, 1992; Yamanaka, 1959) and have ranges extending from northern to western Japan. The 12 species include five conifers (*Abies veitchii* Lind., *Picea jezoensis* (Siebold et Zucc.) Carrière, *Pinus koraiensis*., *Thuja standishii* (Gordon) Carrière and *Tsuga diversifolia*) and 7 angiosperms (*Acer ukurunduense* Trautv. et C.A.Mey., *Berberis amurensis* Rupr., *Betula ermanii* Cham., *Ilex rugosa* F.Schmidt, *Oplopanax japonicus* Nakai, *Rhododendron brachycarpum* D.Don ex G.Don and *Vaccinium vitis‐idaea* L.). Four (*A. veitchii*, *O. japonicus*, *T. diversifolia* and *T. standishii*) are endemic to Japan while the remaining eight species occur in other parts of Eurasia including Korea, northeast China and Far East Russia (Krestov & Nakamura, 2002; Song, 1991). For 8 of 12 species, the whole chloroplast genome is available but these consist of single samples collected from their ranges outside Japan (e.g. in China, *B. ermanii* and *A. ukurunduense*, and South Korea, *B. amurensis*, *V. vitis‐idaea*, *P. koraiensis* and *P. jezoensis*) or in Japan, *T. diversifolia* and *T. standishii*. No whole chloroplast genome is available for *O. japonicus*, *R. brachycarpum*, *I. rugusa* or *A. veitchii*. Here, we report intraspecific chloroplast genome diversity (single nucleotide polymorphisms (SNPs) and indels including simple sequence repeats (SSRs)) based on whole chloroplast genome sequencing of these species for the first time, identify how this intraspecific diversity is geographically distributed and, lastly, assess how the diversity identified in this study is related to already published genomes of the same species.

**TABLE 1:** Details of the 12 subalpine plants investigated in this study including their taxonomy, distribution and habit.

Species	Family	Distribution	Habit
*Abies veitchii*	Pinaceae	Japanese endemic	Tree
*Acer ukurunduense*	Sapindaceae	Japan/Korea/Far E. Russia/N.E. China	Tree
*Berberis amurensis*	Berberidaceae	Japan/Korea/Far E. Russia/N.E. China	Shrub
*Betula ermanii*	Betulaceae	Japan/Korea/Far E. Russia/N.E. China	Tree
*Ilex rugosa*	Aquifoliaceae	Japan/Far E. Russia	Shrub
*Oplopanax japonicus*	Araliaceae	Japanese endemic	Shrub
*Picea jezoensis*	Pinaceae	Japan/Korea/Far E. Russia/N.E. China	Tree
*Pinus koraiensis*	Pinaceae	Japan/Korea/Far E. Russia/N.E. China	Tree
*Rhododendron brachycarpum*	Ericaceae	Japan/Korea/Far E. Russia	Shrub
*Thuja standishii*	Cupressaceae	Japanese endemic	Tree
*Tsuga diversifolia*	Pinaceae	Japanese endemic	Tree
*Vaccinium vitis‐idaea*	Ericaceae	Northern hemisphere	Shrub

## 2 MATERIALS AND METHODS

### 2.1 Sampling and DNA extraction

Leaf samples from 4 to 12 individuals of each of the 12 study species were collected from across their ranges in Japan and, where possible, from where they occur outside Japan such as N.E. China, South Korea and Far East Russia (for a list of all samples, see Table S1). In Japan, the sampling included areas where the species are widespread in central Honshu but also the most northern populations in Hokkaido/Tohoku and most southern parts of the species range in western Japan. DNA was extracted using either the DNeasy Plant Mini Kit (Qiagen) or a modified CTAB protocol. DNA concentration and quality were assessed by agarose gel electrophoresis and a Qubit 2.0 fluorometer (Life Technologies).

### 2.2 Chloroplast genome assembly

Whole genomic DNA was sent to the Beijing Genomic Institute where short‐size libraries were constructed, and paired‐end sequencing (2 × 150 bp except for *T. diversifolia* which were 2 × 100 bp) was performed on an Illumina HiSeq2000 or DNBSEQ‐T7 Genome Analyser. Chloroplast genomes were assembled using GetOrganelle v1.7.7.0 (Jin et al., 2020) and its dependencies including SPAdes (Bankevich et al., 2012) and Bowtie2 (Langmead & Salzberg, 2012), using default setting, or, in the case of *Abies veitchii*, Novoplasty 4.3.1 (Dierckxsens et al., 2016) was used as it was found to perform more reliably for this species. The previously published whole chloroplast genome of *Tsuga diversifolia* (GenBank accession: MH171102) was re‐assembled using GetOrganelle. The chloroplast genomes were annotated using GeSeq (Tillich et al., 2017) with annotations checked by aligning to reference genomes and prepared for submission to GenBank using GB2sequin (Lehwark & Greiner, 2019).

The chloroplast genomes of *R. brachycarpum* and *V. vitis‐idaea* (both Ericaceae) could not be assembled using GetOrganelle or Novoplasty despite trying a range of settings. This was likely caused by an unsolvable tangled graph due to long repeats (Fu et al., 2022) with Ericaceae chloroplast genomes being characterised by gene rearrangement, repetitive regions and IR expansion (Li et al., 2020) making them difficult to assemble using short Illumina reads alone. Instead reads were mapped to reference chloroplast genomes with ‘bwa mem’ (Li, 2013). *Vaccinium vitis‐idaea* reads were mapped to a *V. vitis‐idaea* whole chloroplast genome (sourced from a plant cultivated in Canada, DNA Data Bank of Japan accession number: BR002395) assembled from Oxford Nanopore (SRR25468450) and Illumina NovaSeq reads (SRR25477290) (Hirabayashi et al., 2023) using the ptGAUL pipeline (Zhou et al., 2023) (https://github.com/Bean061/ptgaul). Before chloroplast genome assembly, Nanopore reads were filtered by Filtlong (https://github.com/rrwick/Filtlong) with parameters of ‘‐‐min_length 1000 ‐‐keep_percent 90,’ while Illumina reads (1 Gbp for each pair) were downsampled by the ‘sample’ subcommand of seqtk (https://github.com/lh3/seqtk) ver. 1.4‐r122 with the parameter of ‘‐s 0’. A chloroplast genome of the same species from Korea (GenBank accession: LC521968) was not used because it was found to be missing approximately 5000 bp of sequence. For *R. brachycarpum*, no reference of the same species or raw data including long nanopore reads suitable to assemble a reference genome were available. Therefore, the reads were mapped to the closest available whole chloroplast genome on GenBank as determined by blasting the longest chloroplast contigs produced by GetOrganelle. This resulted in the chloroplast genome of *R. calophytum* Franch (OM373082.1 (Ma et al., 2022)) and *R. shanii* W.P. Fang (MW374796 (Yu et al., 2022)) being the closest. bcftools ver. 1.17 (Li, 2011) was used to create consensus sequences of each mapping incorporating both SNPs and indel sites, whereby the mapping consensus had the reference sequence in regions where the minimum read depth was below 20 or the maximum depth above the average depth + 1 standard deviation. The read mapping‐based assemblies were not annotated or submitted to GenBank due to the relatively high number of ambiguous sites compared to the de novo method and the incorporation of reference sequence in regions with minimum/maximum read depths over the designated thresholds. Rather, they were used primarily for uncovering SNPs and discovery of the main lineages in these species.

### 2.3 Phylogenetic analyses

For the 10 species where whole chloroplast de novo assembly was successful, the intraspecific phylogenetic relationships of the chloroplast genomes were investigated using maximum likelihood implemented in RAxML v.8.2.10 (Stamatakis, 2014). The input files were prepared in Geneious 9.15 (Biomatters, New Zealand) where the whole chloroplast genomes were aligned using MAFFT multiple aligner Version: 1.3.6 (Katoh et al., 2002). Complete published chloroplast genomes of the same species (if available) and, for use as outgroups, up to three complete genomes of different species within the same genus as determined by Blastn (Altschul et al., 1990) (i.e. the three with the closest percentage identity) were included in the alignment. This was done due to the fact that in some cases, the chloroplast genomes of the most closely related species were unavailable and, for the angiosperms, the fact that chloroplast relationships do not necessarily follow species relationships (e.g. for *Betula* (Palme et al., 2004) and *Acer* (Saeki et al., 2011)). Before alignment, one inverted repeat region was removed for all angiosperms and poorly aligned regions were excluded using Gblocks 0.91b (Castresana, 2000) run with default settings except with gaps allowed. The same method as above was used for phylogenetic tree construction for species assembled by reference mapping, *R. brachycarpum* and *V. vitis‐idaea*, except that no outgroups were used.

The accuracy of the whole chloroplast genome sequencing was checked using Sanger sequencing of variations identified in six species (three angiosperms and three conifers), five of whose chloroplast genome was obtained via GetOrganelle de novo method and *R. brachycarpum* obtained using reference mapping. A total of 25 primer pairs in six species were designed around variable sites identified in the whole chloroplast genome data using Primer3 2.3.4. plugin (Untergasser et al., 2012) in Geneious and Sanger sequenced using Supredye (MS Techno Systems) following the manufacturer's instructions for two samples per species. Sequences were determined via capillary electrophoresis on an ABI3130 Genetic Analyzer (Life Technologies, Waltham, MA, USA).

### 2.4 Genetic diversity

The whole chloroplast genomes of each species were aligned in Geneious using MAFFT (Katoh et al., 2002), including accessions of the same species available on GenBank where available (except *Pinus koraiensis* (NC_004677) and *Thuja standishii* (KX832627) where the records on GenBank were found to have unusually high genetic distance from other whole chloroplast genomes of the same species obtained from GenBank and/or this study and were therefore excluded).

The number of non‐informative sites (singletons), parsimony informative sites (i.e. sites that are present at least twice and are therefore potentially informative for phylogenetic analyses), overall nucleotide diversity and average number of nucleotide differences (*k*) were calculated in DNAsp v.6.12.03 (Rozas et al., 2017). The number of indel events and indel diversity was calculated using the multi‐allelic gap option in the same program. Calculations were done separately for those samples from Japan and for all samples (i.e. individuals sampled for this study both within and outside Japan and obtained from GenBank). In addition, the number of SNPs and nucleotide diversity of genes and intergenic spacers across the whole chloroplast genomes of each species was calculated in DNAsp v.6.12.03 using the multi‐domain function.

Due to the presence of ambiguous sites in the reference mapping‐based chloroplast genome assemblies of *R. brachycarpum* and *V. vitis‐idaea* that are not compatible with DNAsp v.6.12.03, we used PopART (Leigh & Bryant, 2015) to calculate the number of SNPs, parsimony informative sites and overall nucleotide diversity.

### 2.5 Chloroplast microsatellite identification

For the 10 species where whole chloroplast de novo assembly was successful, chloroplast microsatellite regions were searched for using Find Polymorphic SSRs which uses the Phobos Tandem Repeat Finder (Mayer, 2008) in Geneious with a repeat unit length of 1–3 bp, a minimum length of 10 bp and a requirement that they occur in all sequences in the alignment.

## 3 RESULTS

### 3.1 Chloroplast genome assembly

An average of 8,285,799 reads were obtained per sample for the 12 species (Table S2). For the 10 species that had their whole chloroplast genome successfully de novo assembled, the percentage of total reads that were from the chloroplast genome varied between 0.24% and 6.64% (Table S2) with substantial difference between angiosperms, average of 3.46%, and the large nuclear genome bearing conifers with an average of 0.90%. As a consequence, overall read coverage of the chloroplast genome was 266.0 for angiosperms to 92.8 for conifers (Table S2). Whole chloroplast genome lengths were between 156,109 and 166,708 bp for angiosperms and between 116,924 and 130,925 bp for conifers. The whole chloroplast genomes of each species did not differ substantially in length (average difference = 185.8 bp) with the most similar being *I. rugosa* (6 bp maximum difference between samples) and the most different being *B. amurensis* (431 bp maximum difference). All sequences obtained from direct Sanger sequencing were identical to the chloroplast genomes (except for some ambiguous sites in the read mapping consensuses of *R. brachycarpum*) and confirmed all the variable sites. For the list of GenBank accession numbers for each sample, see Table S2, and for *R. brachycarpum* and *V. vitis‐idaea* sequence data, see the fasta alignments in the Supplementary Materials.

### 3.2 Genetic diversity of de novo‐assembled species

An average of 56.6 SNPs per species were discovered when considering only Japanese samples (Table 2) and 76.8 when including all samples (Table 2 and Table S3). A total of 31% of all SNPs were parsimony informative for Japan and 41% for all samples. For both angiosperms and conifers, the number of SNPs for samples from Japan varied greatly between species with highest values of 138 for *B. amurensis* and 110 for *P. jezoensis* and lowest being 2 for *T. standishii* and 25 for *I. rugosa*, respectively (Table 2). When considering all samples, the number of SNPs increased by between 6.4 and 1.08 times (average 2.21 times) with the highest increase in *A. ukurunduense* and *P. jezoensis* (Table S3). An average of 61.5 indels events were observed in Japanese samples with a maximum of 147 in *B. ermanii* and a low of 17 in *T. standishii*. Similar to SNPs, the number of indel events increased when including all samples (average 83.8 per species). Considering SSR‐type indels for Japanese samples, mono‐repeats were more common than either di‐ or tri‐repeats in all species with an average of 42.8 per species ranging from 72 in *B. amurensis* to 22 in *T. standishii* (Table 3). This compares to an average per species of 9.3 for di‐ and 6.4 for tri‐repeats. An average of 54.9% of mono‐repeats were polymorphic varying from 90.2% in *A. veitchii* to 0% in *T. standishii*. This contrasts with an average of 7.5% and 4.7% per species being polymorphic for di‐ and tri‐repeats respectively. When considering all samples, the number of overall mono‐, di‐ and tri‐repeats increased slightly with the number of polymorphic ones increasing to 59.7%, 8.06% and 7.4% for mono‐, di‐ and tri‐ repeats, respectively (Table S4).

**TABLE 2:** Summary of genetic diversity identified in the 12 study species including single nucleotide polymorphisms and indels based on the Japan dataset.

Species	No. samples	Total sites	Sites (excluding gaps)	Monomorphic sites	Singletons	Parsimony informative sites	Nucleotide diversity (*Pi*)	Average number of nucleotide differences (*k*)	Indel events	Indel diversity per site (*Pi*(*i*))
*Acer ukurunduense*	6	130,025	129,681	129,671	3	7	0.00004	4.7	31	0.00011
*Berberis amurensis*	8	129,781	128,946	128,808	95	43	0.00036	46.1	139	0.00041
*Betula ermanii*	8	134,983	134,079	134,008	47	24	0.00018	24.4	147	0.00039
*Ilex rugosa*	9	131,506	131,476	131,451	17	8	0.00006	7.6	26	0.00008
*Oplopanax japonicus*	8	130,338	130,185	130,125	6	54	0.00022	29	29	0.0001
*Abies veitchii*	7	121,579	121,109	121,046	57	6	0.00016	19.8	100	0.00031
*Picea jezoensis*	4	124,559	124,047	123,937	103	7	0.00046	56.5	53	0.00022
*Pinus koraiensis*	4	117,154	116,760	116,731	26	3	0.00013	15	37	0.00017
*Thuja standishii*	5	131,166	130,225	130,223	2	0	0.000006	0.8	17	0.00006
*Tsuga diversifolia*	4	121,291	120,827	120,769	34	24	0.00027	33	36	0.00017
*Rhododendron brachycarpum* ^a^	7	157,211	–	157,122	47	42	0.00023	–	–	–
*Rhododendron brachycarpum* ^b^	7	155,954	–	155,875	46	33	0.00019	–	–	–
*Vaccinium vitis‐idaea* ^c^	10	144,322	–	144,109	129	84	0.00043	–	–	–

*Note*: Total sites analysed is the length of the whole chloroplast genome after excluding one inverted repeat.

^a^ Mapped to MW374796.

^b^ Mapped to OM373082.

^c^ Mapped to the *V. vitis‐idaea* whole chloroplast genome assembled from the Oxford Nanopore and Illumina NovaSeq reads of Hirabayashi et al. (2023).

**TABLE 3:** Summary of genetic diversity identified at simple sequence repeat (SSR) regions identified in the de novo‐assembled whole chloroplast genomes of 10 study species for Japan samples.

Species	No. samples	No. polymorphic SSRs (mono‐repeats >9 length)	No. monomorphic SSRs (mono‐repeats >9 length)	No. polymorphic SSRs (di‐repeats >9 length)	No. monomorphic SSRs (di‐repeats >9 length)	No. polymorphic SSRs (tri‐repeats >9 length)	No. monomorphic SSRs (tri‐repeats >9 length)
*Acer ukurunduense*	6	21	47	0	3	0	12
*Berberis amurensis*	8	53	19	0	9	2	2
*Betula ermanii*	8	44	17	2	16	1	11
*Ilex rugosa*	9	21	30	0	4	0	5
*Oplopanax japonicus*	8	9	18	0	7	0	2
*Abies veitchii*	7	37	4	1	13	0	9
*Picea jezoensis*	4	17	8	4	7	0	5
*Pinus koraiensis*	4	20	16	0	6	0	0
*Thuja standishii*	5	0	22	0	14	0	9
*Tsuga diversifolia*	4	13	12	0	7	0	6

Based on Japan‐only data, an average of 85.2% of regions (genes and intragenic spacers) in the angiosperm chloroplast and 86.8% of regions in the conifer chloroplast were invariable with most of the variable regions only having one SNP (Figures S1 and S2). The results for all samples are not shown because they are nearly identical to those based on Japan‐only data. For the majority of species, the most diverse regions based on nucleotide diversity were intragenic spacers except for the low‐diversity *A. ukurunduense* in Japan where half of the most diverse regions were within genes (Tables 4 and 5). The most diverse regions based on nucleotide diversity were particular to each species in 56 cases, 13 regions were found in at least 2 species and only 3 regions, trnH‐GUG—psbA, ycf1—ndhF and the long ycf1 gene, were observed in 3 species and none in 4 or more. For the results based on all samples, see Table S5.

**TABLE 4:** The top 10 most variable regions of the de novo‐assembled whole chloroplast genome of Japanese samples of five angiosperm study species according to nucleotide diversity (*Pi*).

No.	*Acer ukurunduense*	*Berberis amurensis*	*Betula ermanii*	*Ilex rugosa*	*Oplopanax japonicus*
1	*rpl33—rps18*	*trnH‐GUG—psbA*	*ycf1—ndhF*	*ndhG—ndhI*	*ycf1—ndhF*
2	*psbB—psbT*	*psbI—trnS‐GCU*	*psbZ—trnG‐GCC*	*psbI—trnS‐GCU*	*rps2—rpoC2*
3	*ndhC—trnV‐UAC*	*ycf1—ndhF*	*ndhD—psaC*	*trnH‐GUG—psbA*	*psbZ—trnG‐GCC*
4	*trnS‐GCU—trnG‐UCC*	*trnL‐UAG—ccsA*	*rps15—ycf1*	*rps4—trnT‐UGU*	*rpl16—rps3*
5	**atpF**	*atpI—rps2*	**ycf1**	*trnG‐GCC—trnfM‐CAU*	*trnG‐GCC— trnfM‐CAU*
6	**petA**	*ndhF—rpl32*	**rps15**	*psbB—psbT*	*psbC—trnS‐UGA*
7	**clpP1**	**accD**	*petG—trnW‐CCA*	*trnS‐UGA—psbZ*	*ndhE—ndhG*
8	**ycf1**	*trnS‐GGA—rps4*	*ndhF—rpl32*	*rps2—rpoC2*	*trnH‐GUG—psbA*
9	**ndhA**	*trnN‐GUU—ycf1*	**rpl14**	*psbA—matK*	*rps8— rpl14*
10	–	*trnQ‐UUG—psbK*	**cemA**	**psbA**	*rps18—rpl20*

*Note*: Intergenic spacers are italicised while genes are shown in bold. Note that *Acer ukurunduense* only had nine variable regions.

**TABLE 5:** The top 10 most variable regions of the de novo‐assembled whole chloroplast genome of Japanese samples of the five conifer study species according to nucleotide diversity (*Pi*).

No.	*Abies veitchii*	*Picea jezoensis*	*Pinus koraiensis*	*Thuja standishii*	*Tsuga diversifolia*
1	*ycf2—trnH‐GUG*	*rpoA—rps11*	*rps12—rps7*	*ndhJ—rps12*	clpP—ycf12
2	*rpl33—psaJ*	**trnR—UCU**	*trnfM‐CAU—trnG‐GCC*	*chlB—matK*	*rps12—rps7*
3	*atpE—trnM‐CAU*	**trnS—UGA**	**psbK**	–	*rps11—rpl36*
4	*trnl‐CAU—psbA*	*rpoC1—rpoC2*	*rrn5—trnR‐ACG*	–	*psbJ—psbL*
5	*psaJ—trnP‐UGG*	*chlB—trnQ‐UUG*	*trnS‐GCU—psaM*	–	*ycf2—trnH‐GUG*
6	*trnP‐UGG—trnW‐CCA*	*ycf12—clpP*	*trnQ‐UUG—psbK*	–	*chlN—ycf1*
7	*clpP—rps12*	*trnH‐GUG—trnT‐GGU*	*rpl23—trnI‐GAU*	–	**rps15**
8	**psbK**	*psaJ—trnP‐UGG*	*trnT‐GGU—trnS‐GCU*	–	**rpl14**
9	*trnM‐CAU—trnV‐UAC*	*ycf12—psbB*	*psbK—psbI*	–	*psbK—psbI*
10	rpl23—trnV‐GAC	*ycf1—rps15*	*chlN—rps15*	–	**ycf1**

*Note*: Intergenic spacers are italicised while genes are shown in bold. Note that *Thuja standishii* only had two variable regions.

### 3.3 Genetic diversity of reference mapping assembled species

The number of overall SNPs identified in *V. vitis‐idaea* was 213 for Japanese samples with 84 being parsimony informative. For all samples, the number of SNPs increased to 238 with 84 parsimony informative. In *R. brachycarpum*, the mapping using the *R. shanii* reference chloroplast genome (MW374796) resulted in a lower number of overall SNPs but higher number of parsimony informative SNPs being uncovered (89/107 overall SNPs with 42/54 parsimony informative for Japan and all samples, respectively) compared to mapping to *R. calophytum* (OM373082) (79/186 overall SNPs with 33/44 parsimony informative for Japan and all samples, respectively).

### 3.4 Phylogenetic relationships

**FIGURE 1:**
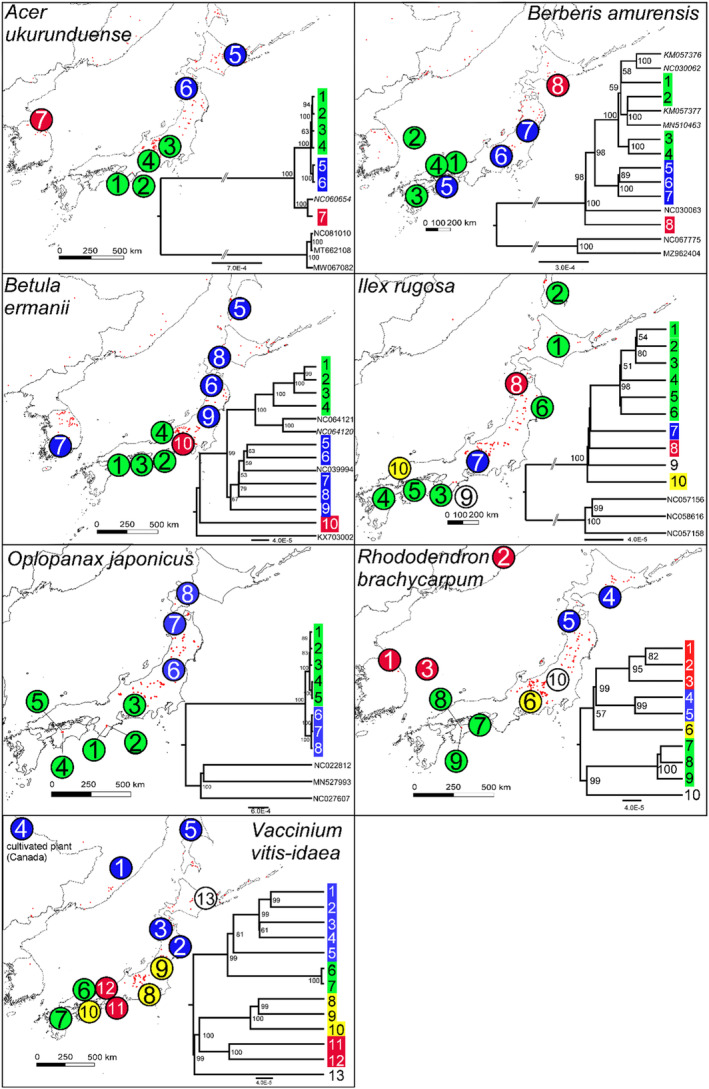
Distribution of the samples, indicated by numbered circles, used for whole chloroplast genome sequencing of the seven investigated angiosperm species included in this study. The colours indicate their respective clades in the maximum‐likelihood phylogenetic tree with bootstrap values above 50% shown. Whole chloroplast genomes sourced from GenBank are indicated by the GenBank accession numbers with those in italics conspecific accessions and non‐italicised ones outgroup accessions. The results for *Rhododendron brachycarpum* are based on the reference mapping to *R. shanii* (MW374796) while for *Vaccinium vitis‐idaea*, the results are based on reference mapping to the whole chloroplast genome of a cultivated individual of *Vaccinium vitis‐idaea* from Canada. The red dots indicate the species' modern distribution. The distribution records were compiled from the Global Biodiversity Information Facility (www.gbif.org) and personal records. Records of all contributing GBIF datasets are as follows: *Acer ukurunduense* (https://doi.org/10.15468/dl.2xz63r), *Berberis amurensis* (https://doi.org/10.15468/dl.nfx96f), *Betula ermanii* (https://doi.org/10.15468/dl.us2k4d), *Ilex rugosa* (https://doi.org/10.15468/dl.b4jub4), *Oplopanax japonicus* (https://doi.org/10.15468/dl.46desu), *Rhododendron brachycarpum* (https://doi.org/10.15468/dl.zkg6e2) and *Vaccinium vitis‐idaea* (https://doi.org/10.15468/dl.avmf3u).

**FIGURE 2:**
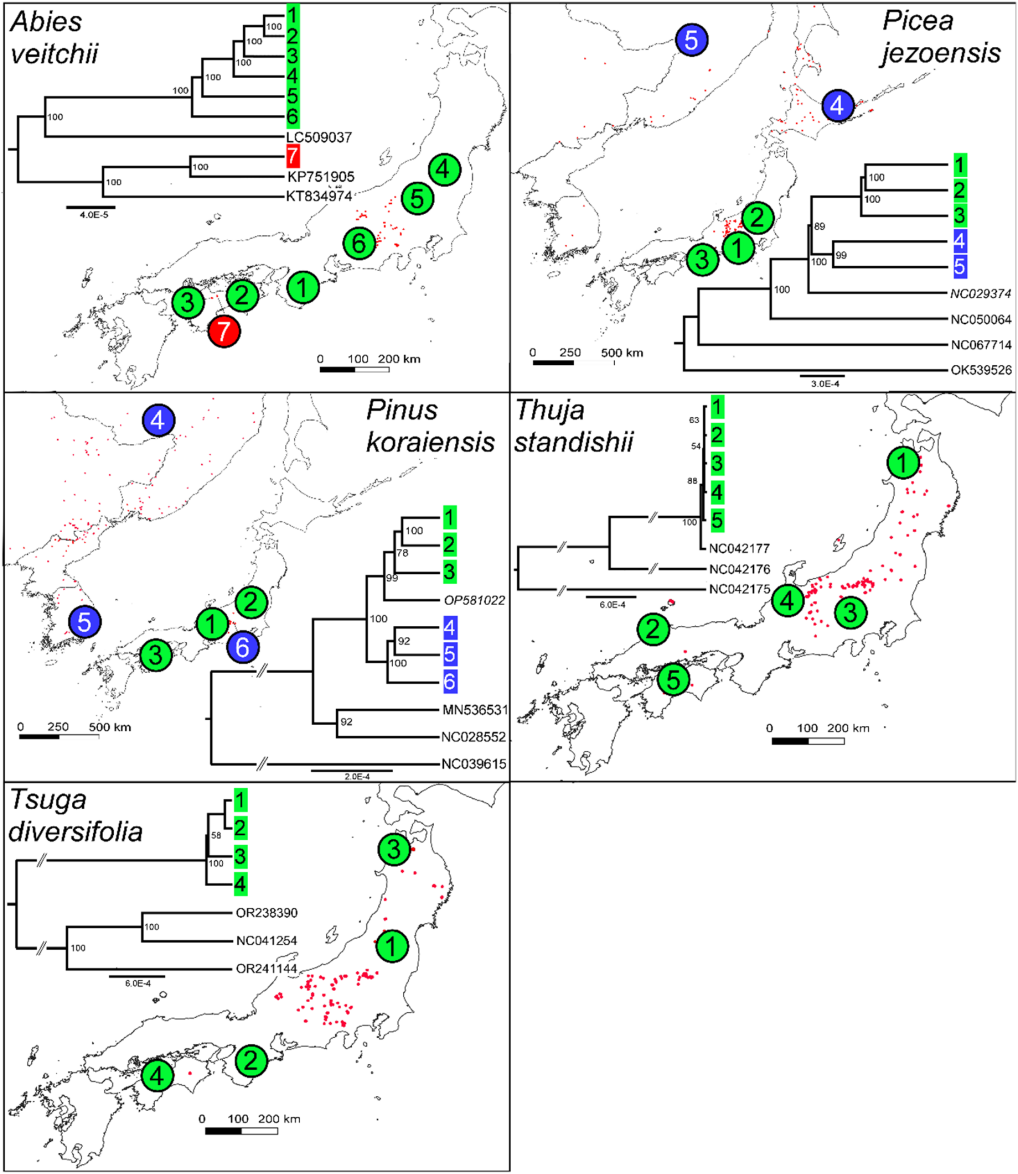
Distribution of the samples, indicated by numbered circles, used for whole chloroplast genome sequencing of the five investigated conifer species included in this study. The colours indicate their respective clades in the maximum‐likelihood phylogenetic tree with bootstrap values above 50% shown. Whole chloroplast genomes sourced from GenBank are indicated by the GenBank accession numbers with those in italics conspecific accessions and non‐italicised ones outgroup accessions. The red dots indicate the species' modern distribution. The distribution records were compiled from the Global Biodiversity Information Facility (www.gbif.org) and personal records. Records of all contributing GBIF datasets are as follows: *Abies veitchii* (https://doi.org/10.15468/dl.rfhzbt), *Picea jezoensis* (https://doi.org/10.15468/dl.hykecj), *Pinus koraiensis* (https://doi.org/10.15468/dl.dxjx32), *Thuja standishii* (https://doi.org/10.15468/dl.lbuhvp) and *Tsuga diversifolia* (https://doi.org/10.15468/dl.2grpa5).

The chloroplast variation in most species was found to be distributed in well‐supported clades with clear non‐overlapping geographical ranges (see Figures 1 and 2). For example, *B. ermanii*, *A. ukurunduense*, *O. japonicus*, *P. jezoensis*, *R. brachycarpum*, *V. vitis‐idaea* and (to a less clear extent) *B. amurensis* were found to harbour northern and southern distributed lineages. These lineages coincided with the distribution of two varieties in the case of *P. jezoensis*, var. *jezoensis* distributed in Far East Russia, northeast China and Hokkaido and var. *hondoensis* distributed in Honshu, Japan (Aizawa et al., 2007). For *R. brachycarpum*, phylogenetic relationships were similar between the results based on the two references used for read mapping with all main clades recovered in both but the phylogeny based on the *R. calophytum* reference was less well resolved in the early diverging branches probably due to a lower number of SNPs recovered (Figure 1 and Figure S3). On the other hand, the lineages identified in *P. koraiensis* and *I. rugosa* had overlapping ranges, while for *A. veitchii*, a diverged lineage was observed in only one individual at the southern edge of the species range (Figures 1 and 2). For both *T. diversifolia* and *T. standishii*, all individuals harboured genetically similar chloroplast genomes with no diverged lineages (Figure 2). GenBank accessions of the same species were mostly placed within one of the distinct clades identified in this study (e.g. *P. koraiensis*, A. *ukurunduense* and *B. amurensis*). In three species, *A. veitchii*, *B. ermanii* and *B. amurensis*, GenBank accessions of outgroup species were nested within identified clades (Figures 1 and 2).

## 4 DISCUSSION

This is one of the first studies to develop ultra‐barcodes for species representing a distinct biome and contributes to an increasing trend to assemble whole chloroplast genomes for genetic resource development studies. For example, Song et al. (2023) included whole chloroplast genomes, along with traditional short Sanger‐based fragments, in a barcode library of the flowering plants of arid NW China while Krawczyk et al. (2018) used whole chloroplast genomes in a barcode library for the genus *Stipa*. However, studies investigating *within*‐species variation of the whole chloroplast genome remain rare. In this study, the assembly of whole chloroplast genomes from short‐read genome skimming data facilitated the discovery of significant levels of intraspecific chloroplast variation in important 12 subalpine forest trees and shrubs. With the exception of *T. standishii*, this includes tens of SNPs, indels and polymorphic simple sequence repeat regions within each species. A total of 31% and 41% of SNPs were parsimony informative for Japan and all samples respectively. These will be useful for elucidating genetic relationships and divergence of populations across the range of these 12 subalpine forest species. However, hotspots of variation were exceedingly rare with the vast majority of variable genes or intragenic regions only having a single SNP.

While the whole chloroplast genomes of 10 species were efficiently and accurately assembled using de novo assembly‐based methods, this method failed using short reads for the two Ericaceae species, *R. brachycarpum* and *V*. *vitis‐idaea*. However, we show that mapping reads to a reference even in the absence of a con‐specific reference in the case of *R. brachycarpum* can provide a reliable assay of chloroplast variation. For both species, tens of SNPs and distinct geographically based chloroplast lineages were identified.

### 4.1 Potential applications in phylogeography and conservation genetics

By identifying intraspecific chloroplast SNPs, indels and SSRs, this study will accelerate phylogeographic and conservation genetic studies of these 12 important subalpine forest species. The whole chloroplast genome approach to identifying intraspecific chloroplast variation has distinct advantages over using methods based on previously published universal primers. Firstly, it maximises the ability to identify intraspecific chloroplast variation, whether it consists of rare singleton SNPs to deeply diverged lineages (Wang et al., 2023; Worth et al., 2021), in a genome that, as this study demonstrates, is mostly invariable at the species level (sites with SNPs comprised between 0.0015% and 0.106% of all sites in the 10 species where whole chloroplast genomes were assembled via de novo methods for Japan samples). In fact, any variation, especially potentially phylogenetically informative sites, was found to be scattered widely apart across the genome and therefore are not guaranteed to be captured via traditional Sanger‐based methods. Secondly, primer pairs can be designed to target fragments that ensure the most efficient screening of chloroplast variation (both singletons and parsimony informative SNPs) according to each project's resources and objectives. Thirdly, simultaneously, chloroplast SSRs, which have a faster rate of evolution than other types of chloroplast polymorphism (Provan et al., 1999) and are particularly sensitive markers for assessing population size changes and genetic diversity (Provan et al., 2001), can be easily identified. Lastly, the method can reveal unexpected patterns of chloroplast sharing with congeneric species as demonstrated by the nesting of GenBank accessions of related species of *Abies*, *Betula* and *Berberis* in the intraspecific variation found in Japan of *A. veitchii*, *B. ermanii* and *B. amurensis*.

The identification of areas with unique genetic lineages (i.e. evolutionary significant units (Moritz, 1994)) and/or high levels of genetic diversity could help to prioritise allocation of limited conservation resources and inform management decisions for the 12 subalpine species. This chloroplast information could also be used to identify seed source zones for reforestation or translocation (Tsumura, 2022), which is particularly important given the overall threat of decline in subalpine forests under global warming, deer browsing and the small and isolated nature of some populations. For example, *Ilex rugosa* has only one population on the island of Kyushu and one in the whole of the Chugoku area of western Japan, while the declining isolated population of *Oplopanax japonicus* on the Kii Peninsula depends almost entirely on the protection of deer‐proof fences for its persistence. However, in all these cases, nothing is known about the divergence and genetic diversity of these populations. Range‐wide studies are required to clarify the distribution of chloroplast variation identified in this study, including the significance of apparently southern versus northerly distributed distinct lineages identified in some of the 12 species.

### 4.2 Genetic markers for ancient and environmental DNA studies

The whole chloroplast genome ultra‐barcodes assembled in this study will be useful for various applications involving the identification of species, and within‐species genetic variation, from DNA in modern and ancient samples. These include investigations with solely scientific objectives or with importance for human health, forensics and environmental monitoring (Setsuko et al., 2023). Indeed, because of the high copy number of chloroplast genomes, chloroplast DNA fragments are more readily isolated from modern and ancient DNA samples than nuclear fragments (Lennartz et al., 2021) making the chloroplast particularly applicable to studies based on environmental samples.

One of the most impactful contributions that the chloroplast genomes assembled in this study could make is in the study of palaeoecology. Fossils of subalpine plants including most of the 12 species investigated in this study are found in Last Glacial age sediments across Japan where they are sometimes abundant (e.g. Nishiuchi et al., 2017). Ancient DNA studies utilising the ultra‐barcodes assembled in this study would enable the independent assessment of past species diversity and abundance, for example, via methods such as sedaDNA (Liu et al., 2020), to contrast with solely fossil (macro‐ and/or micro‐fossils)‐based conclusions. In Japan such an approach may enable the identification of morphologically similar but ecologically diverged species that have been difficult to distinguish based on fossil morphology alone, for example, when only fossil pollen is available and/or when macrofossils lack informative parts such as reproductive structures. In Japan, conifers of the genera *Tsuga*, *Abies* and *Pinus* have both temperate and subalpine representatives while *Picea* has both geographically restricted and widespread representatives. Therefore, the ability to distinguish species of these genera using ancient DNA would have large implications for our understanding of past vegetation and migration/range contraction histories of specific forest biomes in Japan.

Whole chloroplast genomes are particularly promising for ancient and environmental DNA studies of conifers given the high level of species divergence at the paternally inherited chloroplast in conifers (Mogensen, 2009). In addition, having the whole chloroplast genome available means that any chloroplast genome fragment obtained from modern or ancient samples using next‐generation sequencing methods, typically comprising small fragments under 50 bp in ancient DNA studies (Parducci et al., 2019), will likely be matchable to some part of the genome and, depending on the length and diversity of the fragment (and the level of chloroplast sharing in the case of angiosperms), the species will likely be identified. The likelihood of accurate species identification will only improve with increasing number of different chloroplast fragments recovered. In Japan, increasing the number of whole chloroplast genomes available, especially for species‐rich genera (e.g. *Picea*), is crucial to increasing the potential and accuracy of such DNA studies. Knowledge about the intra‐specific chloroplast lineages in each species may also provide an opportunity to investigate the past distribution of specific lineages.

## AUTHOR CONTRIBUTIONS

**James R. P. Worth:** Conceptualization (lead); data curation (lead); formal analysis (lead); funding acquisition (lead); investigation (lead); methodology (lead); software (lead); validation (lead); visualization (lead); writing – original draft (lead); writing – review and editing (lead). **Satoshi Kikuchi:** Investigation (supporting); resources (supporting); writing – review and editing (supporting). **Seiichi Kanetani:** Resources (supporting); writing – review and editing (supporting). **Daiki Takahashi:** Resources (supporting); writing – review and editing (supporting). **Mineaki Aizawa:** Resources (supporting); writing – review and editing (supporting). **Elena A. Marchuk:** Resources (supporting); writing – review and editing (supporting). **Hyeok Jae Choi:** Funding acquisition (supporting); resources (supporting); writing – review and editing (supporting). **Maria A. Polezhaeva:** Resources (supporting); writing – review and editing (supporting). **Viktor V. Sheiko:** Resources (supporting); writing – review and editing (supporting). **Saneyoshi Ueno:** Data curation (supporting); formal analysis (supporting); methodology (supporting); writing – review and editing (supporting).

## CONFLICT OF INTEREST STATEMENT

The authors declare no conflict of interest.

## Supporting information


Appendix S1

Data S1

## Data Availability

All whole chloroplast genomes are available on GenBank for the 10 species whose whole chloroplast genome was assembled using de novo methods. For *R. brachycarpum* and *V. vitis‐idaea* results, see the fasta alignments in the Supplementary Materials. Raw read data have also been deposited in the NCBI sequence read archive (Bioproject: PRJNA1076900).
